# The interaction of perfluoroalkyl acids and a family history of diabetes on arthritis: analyses of 2011–2018 NHANES

**DOI:** 10.1186/s12889-024-17879-2

**Published:** 2024-02-12

**Authors:** Shuting Yang, Xuqi Li, Zhengdong Jiang

**Affiliations:** Department of General Surgery, The First Affiliated Hospital of Xi’an Jiaotong University, 710061 Xi’an, Shaanxi China

**Keywords:** NHANES, Family history of diabetes, Arthritis, Perfluoroalkyl acids

## Abstract

Whether a family history of diabetes (FHD) and exposure to perfluoroalkyl acids (PFAAs) are correlated with an increased risk of developing arthritis remains unclear. This cross-sectional study was conducted to explore the correlations between FHD or exposure to PFAAs and arthritis as well as their interaction using the National Health and Nutrition Examination Survey (NHANES). In total, 6,194 participants aged ≥ 20 years from the 2011–2018 NHANES were enrolled. PFAAs are a cluster of synthetic chemicals, including perfluorononanoic acid (PFNA), perfluorooctanoic acid (PFOA), perfluorooctane sulfonic acid (PFOS), perfluorodecanoic acid (PFDA) and perfluorohexane sulfonic acid (PFHxS). FHD was evaluated using self-reported questionnaires. Arthritis was classified into three types, rheumatoid arthritis (RA), osteoarthritis (OA), and others, which were diagnosed using questionnaires. Generalized linear models (GLMs) were used to test the correlation between FHD and arthritis. To examine the joint effects of PFAAs and FHD on arthritis, interaction terms were applied in the GLM. Arthritis incidence was 26.7% among all participants. FHD was associated with both RA [OR = 1.70 (95% CI: 1.15–2.50)] and other types of arthritis [OR = 1.62 (95% CI: 1.21–2.16)]. However, the relationship between FHD and OA was not significant after adjustment (*P* = 0.18). Interaction outcomes indicated that higher PFDA levels increased the association between FHD and arthritis. FHD is associated with an increased incidence of arthritis, which may be increased by PFDA. Given the heavy burden of arthritis, preventive measures for arthritis and reduction of PFAAs exposure for patients with FHD are required.

## Introduction

Arthritis, defined as a group of rheumatic conditions, including rheumatoid arthritis (RA), osteoarthritis (OA), and other types of arthritis [1], has become one of the most common disabling health conditions in the United States [2]. With chronic pain, activity restriction, and incapacity, arthritis imposes a huge burden on clinical and public health systems [3]. Previous studies have shown that 0.9% of adults in the United States have RA [2] and 6.13% have OA [4]. The prevalence of arthritis is predicted to increase to 78.4 million among adults in the United States by 2040 [5]. Chronic inflammation and immunity may play significant roles in the development of arthritis-related disorders, such as OA and RA [6]. Additionally, metabolic syndrome, genetic abnormalities, age, sex, and eating disorders are influencing factors for arthritis [7]. Nevertheless, it is not clear whether a family history of diabetes (FHD) and perfluoroalkyl acids (PFAAs) also induce arthritis.

A previous study has shown that FHD is independently associated with diabetes [8]. Vornanen et al. revealed that family history information can be considered a screening tool for type 2 diabetes mellitus (T2DM), with substantial clinical validity [9]. There is evidence that people with diabetes are more likely to suffer from arthritis. In particular, Rehling et al. observed that diabetes is correlated with increases in the prevalence of OA and risk of RA [10]. FHD is associated with metabolic diseases, including subclinical atherosclerosis [11], diabetic foot complications [12], and fatty liver disease [13]. However, the correlation between FHD and arthritis has not been established.

Polyfluoroalkyl substances (PFAS), represented by PFAAs, are a large cluster of artificial chemicals, including perfluorononanoic acid (PFNA), perfluorooctanoic acid (PFOA), perfluorooctane sulfonic acid (PFOS), perfluorodecanoic acid (PFDA), and perfluorohexane sulfonic acid (PFHxS) [14]. They are used as ingredients or surface protectors for consumer applications or fire-fighting foams [14, 15]. Exposure to contaminated drinking water, food products, dust, and consumer products containing PFAS causes serum PFAS accumulation [14, 16]. Although the Stockholm Convention has restricted the use and elimination of PFOS and PFOA in 2009 and 2019, they are continuously detected worldwide because of their bioaccumulation and persistence [17]. Moreover, PFOA and PFOS have been detected in 86% and 100% of breast milk samples, respectively, from breastfeeding women [18]. These phenomena indicate that PFAA exposure is a critical, widespread, and urgent problem that needs to be addressed.

To date, the correlations between PFAAs and diabetes as well as some specific types of arthritis have been evaluated. However, conflicting results have been obtained. For example, a recent study has found associations between PFOA and gestational diabetes mellitus (GDM) [19]. However, in a study of Norwegian women, there were no significant associations between PFAA concentrations and the type 2 diabetes (T2D) incidence [20]. Serum PFAA levels are correlated with serum uric acid levels in gout, which is a type of arthritis but different from RA and OA [21, 22]. Moreover, an occupational study revealed that PFAAs are correlated with disease activity in RA [23]. There are associations between serum PFOA concentrations and some immune marker levels, including anti-cyclic citrullinated peptide antibody (ACPA), C-reactive protein (CRP), and immunoglobulin G (IgG) levels, which play critical roles in inflammation and are immunotoxic to humans [23]. Rheumatoid factors (RF) and ACPA bind to IgG Fc fragments [24]; an increased level of IgG may promote the up-regulation of ACPA and RF. However, the effect of PFAAs modification on the correlations between FHD and arthritis is not clear.

In this cross-sectional study, the correlations between FHD or exposure to PFAAs and arthritis and their interaction with arthritis were evaluated using the National Health and Nutrition Examination Survey (NHANES).

## Methods

### Study population

NHANES is a continuous, national survey of the civilian population conducted by the Centers for Disease Control (CDC/NCHS) in the United States to evaluate population health and nutritional status (https://wwwn.cdc.gov/nchs/data/nhanes/analyticguidelines/11-16-analytic-guidelines.pdf and https://www.cdc.gov/nchs/data/series/sr_02/sr02-190.pdf).

Consent was obtained from all adult participants for this survey, and detailed information can be obtained from the website. All interviews, physical tests, and laboratory examinations, including urine and blood collection, were conducted by NCHS-trained professionals.

In this study, after combining public files for the NHANES 2011–2012, 2013–2014, 2015–2016, and 2017–2018 cycles with the recommended methods (https://wwwn.cdc.gov/nchs/data/nhanes/analyticguidelines/11-16-analytic-guidelines.pdf and https://www.cdc.gov/nchs/data/series/sr_02/sr02-190.pdf), we conducted a survey of adults who were not pregnant and had PFAAs information (*n* = 6194) from 2011 to 2018. All participants provided written informed consent, and the NHANES was approved by the NCHS Research Ethics Review Board (Continuation of Protocol #2011-17 http://www.cdc.gov/nchs/nhanes/irba98.htm).

### Assessment of perfluoroalkyl substances, vitamin D, and cholesterol

Blood samples of the participants were collected and analyzed by the NHANES laboratory team. Serum PFAS were examined by the National Center for Environmental Health (NCEH) team with a limit of detection of 0.08 to 0.2 ng/mL; levels of 14 types of PFAS were analyzed. Three PFAAs (PFNA, PFDA, and PFHxS) were investigated in this study (https://wwwn.cdc.gov/nchs/data/nhanes/2015-2016/labmethods/PFAS_I_MET.pdf). Vitamin D was tested via high-performance liquid chromatography-tandem mass spectrometry (HPLC-MS/MS). Cholesterol levels were assessed; direct high-density lipoprotein (HDL)-cholesterol and total cholesterol were both examined using an enzymatic method. The NCEH uses the limit of detection (LOD) divided by the square root of two concentrations for values below the LOD.

### Diagnosis of arthritis, FHD, hypertension, and diabetes

Participants were classified as having arthritis using self-reported questionnaires when they responded “Yes” to the item: “Doctor ever said you had arthritis.” Arthritis types were categorized as “Rheumatoid arthritis (RA),” “Osteoarthritis (OA),” or “Other” based on the question “Which type of arthritis was it?” FHD was obtained from the questionnaire with the question “Close relative had diabetes” in the medical condition questionnaire. Hypertension was diagnosed as a complex and systemic disorder, characterized by increased diastolic blood pressure of ≥ 90 mmHg and/or systolic blood pressure (SBP) of ≥ 140 mmHg [25]. Diabetes was diagnosed based on self-reported questionnaires.

### Covariates

Several potential covariates, such as sociodemographic characteristics, health conditions, socioeconomic variables, and lifestyle habits, were included in this study. Sociodemographic factors included sex (male or female), age (years), and race (non-Hispanic White, non-Hispanic Black, Mexican Americans, or other races). In this study, socioeconomic variables included education status (“High school or below,” “College,” or “College graduate or above”). Lifestyle habits included smoking status (“Every day,” “Some days,” “Not at all,” or “Not recorded”) and drinking alcohol ≥ 4 drinks/day (“Yes,” “No,” or “Not recorded”). Health conditions included body mass index (BMI) and overweight status. BMI was calculated by dividing the measured weight (kg) by the square of height (m^2^) and was obtained from the NHANES physical examination data. Overweight/obesity was defined by a “Yes” response to the item “Doctor ever said you were overweight.”

### Statistical analysis

All analyses were performed using Statistical Product Service Solutions (version 26), and the recommended chemical-specific subsample weights were determined. For descriptive analyses, continuous and categorical variables are reported as means ± standard deviations and count data (percentages), respectively. Chi-squared and Student’s *t*-tests were used to evaluate the differences in continuous and categorical variables between participants with and without FHD and between participants with and without arthritis. Correlations among different PFAS and urine metabolites were tested using a Pearson’s correlation analysis.

Generalized linear models (GLM) were used to evaluate the impact of FHD and PFAAs on arthritis and their interactions with arthritis. Odds ratios (OR) and 95% confidence intervals (95% CIs) for arthritis per interquartile range (IQR) increase in FHD were obtained. First, a crude model was employed without adjustment. Then, we adjusted for sociodemographic variables (sex, age, and race) and socioeconomic variables (education) in Model 1. Furthermore, Model 2 was adjusted for lifestyle habits (smoking status and drinking status). Models 3, 4, and 5 were adjusted for BMI, diabetes, and hypertension, respectively. In addition, we classified arthritis into three types and compared the OR values for each arthritis type to identify whether FHD was associated with an increased prevalence of a specific type. Model 6 was included to adjust for smoking, alcohol, and BMI. Furthermore, Model 7 was used to adjust for diabetes and hypertension. All covariates were selected based on previous research.

We applied three models based to determine the impact of PFAAs on arthritis and their modification of the correlation between FHD and arthritis. First, we analyzed the association between PFAAs and arthritis, without the effect of FHD (Model 8). Then, the same model (Model 9) was evaluated, including an additive term for PFAAs and FHD. Finally, we assessed a model (Model 10) that included an interaction term for PFAAs and FHD to explore the interactive effects.

A two-tailed *P* value of < 0.05 was considered statistically significant for the correlation of FHD / PFAAs and arthritis, and a two-tailed *P* value of < 0.10 was considered significant for the interactions between PFAAs and arthritis [26].

## Results

### Descriptive statistics

**Fig. 1:**
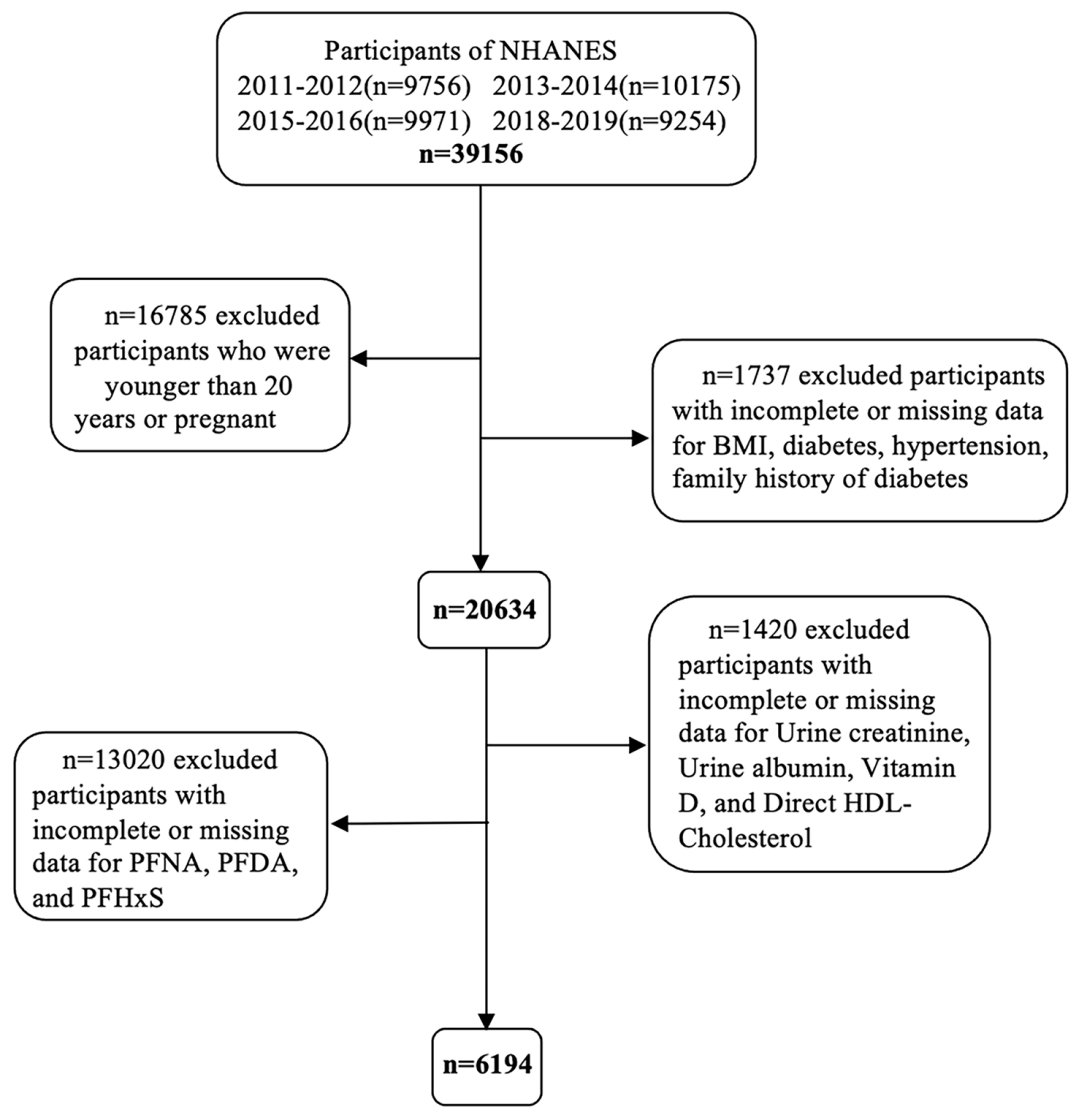
Flow chart of the screening process for the selection of eligible participants in NHANES 2011–2018

In this study, we excluded participants who were younger than 20 years of age, pregnant, or had incomplete or missing data. A detailed flowchart of the participant selection process is presented in Fig. 1. After selection, 6,194 participants were included in this study. Table 1 shows the basic characteristics of the participants in the FHD and non-FHD groups. Overall, 2,698 participants had FHD, with a prevalence of 43.6%. The mean age was 50.28 ± 16.76 years. The mean levels of direct HDL-cholesterol, total cholesterol, and vitamin D were 51.8 mg/dL, 188.93 mg/dL, and 64.53 nmol/L, respectively. Except for the drinking status, a significant correlation was observed between FHD and all characteristics in Table 1.

**Table 1:** Baseline characteristics of the diabetes family history (FHD) group and the non-FHD group

Characteristics ^a^	With FHD n = 2698 (43.6%)	Without FHD n = 3496 (56.4%)	*P*-value
Age (years)	50.28 ± 16.76	48.85 ± 18.02	< 0.01
Sex			
Men	1240 (46.0)	1767 (50.5)	< 0.01
Women	1458 (54.0)	1729 (49.5)	
Education Levels			
High school or below	1228 (45.8)	1486 (42.5)	< 0.01
College	870 (32.2)	1053 (30.1)	
College graduate or above	591 (21.9)	953 (27.3)	
Not recorded	3 (0.1)	4 (0.1)	
Race/Ethnicity			
Non-Hispanic White	879 (32.6)	728 (20.8)	< 0.01
Non-Hispanic Black	712 (26.4)	1053 (30.1)	
Mexican Americans	417 (15.5)	311 (9.5)	
Others	690 (25.5)	1404 (39.6)	
Smoking status			
Every day	426 (15.8)	497 (14.2)	0.01
Some day	123 (4.6)	135 (3.9)	
Not at all	643 (23.8)	766 (21.9)	
Not recorded	1506 (55.8)	2098 (60.0)	
Alcoholic ≥ 4 drinks/day			
Yes	355 (13.2)	407 (11.6)	0.17
No	1798 (66.6)	2350 (67.2)	
Not recorded	1 (0.02)	739 (21.1)	
Arthritis			
RA	180 (6.7)	157 (4.5)	< 0.01
OA	340 (12.6)	359 (10.3)	
Other	339 (12.5)	280 (8.0)	
Without AR	1839 (68.2)	2700 (77.2)	
Hypertension	1142 (42.3)	1128 (32.3)	< 0.01
Diabetes	706 (26.1)	307 (8.8)	< 0.01
Direct HDL-Cholesterol (mg/dL)	51.80 ± 15.55	53.98 ± 16.33	< 0.01
Total Cholesterol (mg/dL)	188.93 ± 42.26	191.04 ± 39.92	< 0.05
Vitamin D (nmol/L)	64.53 ± 29.75	66.07 ± 27.68	< 0.05
BMI	30.64 ± 7.63	28.20 ± 0.08	< 0.01
Overweight	1194 (44.3)	1033 (29.5)	< 0.01

^a^ For continuous variables, numbers represent the mean ± standard deviation, and for categorical variables, numbers represent count (percentage)

Abbreviations: Direct HDL-Cholesterol, direct high-density lipoprotein-cholesterol; BMI, body mass index; FHD, family history of diabetes; RA, Rheumatoid arthritis; OA, Osteoarthritis

Table 2 presents descriptive data according to whether patients had arthritis. Participants with arthritis were further grouped based on the arthritis type. Of all participants with arthritis, 859 (51.9%) had FHD, 890 (25.1%) had diabetes, and 999 (60.4%) had hypertension. The average age was 61.55 years. Apart from the concentrations of direct HDL-cholesterol and total cholesterol, all characteristics displayed in Table 2 showed strong associations in both the AR and non-AR groups.

**Table 2:** Baseline characteristics of the arthritis group and the non-arthritis group

Characteristics^a^	With AR				Without AR n = 4539 (73.3%)	*P*-value
	Total n = 1655 (26.7%)	RA n = 337 (5.4%)	OA n = 699 (11.3%)	Other n = 619 (10.0%)		
Age (years)	61.55 ± 13.58	60.33 ± 13.21	63.10 ± 13.38	60.46 ± 13.86	45.07 ± 16.67	< 0.01
Sex						
Men	647 (39.1)	131 (38.9)	244 (34.9)	272 (43.9)	2360 (52.0)	< 0.01
Women	1008 (60.9)	206 (61.1)	455 (66.1)	347 (56.1)	2179 (48.0)	
Race/Ethnicity						
Non-Hispanic White	759 (45.9)	106 (31.5)	395 (56.5)	242 (39.1)	1516 (33.4)	< 0.01
Non-Hispanic Black	383 (23.1)	122 (36.2)	119 (17.0)	158 (25.5)	1023 (22.5)	
Mexican Americans	180 (10.9)	51 (15.1)	49 (7.0)	80 (12.9) 139	662 (14.6)	
Others	333 (20.1)	58 (17.2)	136 (19.5)	(22.5)	1338 (29.5)	
Education level						
High school or below	816 (49.3)	183 (54.3)	291 (41.7)	342 (55.2)	1907 (42.1)	< 0.01
College	534 (32.3)	112 (33.2)	238 (34.0)	184 (29.7)	1389 (30.6)	
College graduate or above	305 (18.4)	42 (12.5)	170 (24.3)	93 (15.0)	1239 (27.3)	
Alcoholic ≥ 4 drinks/day						
Yes	255 (15.4)	62 (18.4)	101 (14.4)	92 (14.9)	507 (11.2)	< 0.01
No	1085 (65.6)	202 ((59.9)	480 (68.7)	403 (65.1)	3063 (67.5)	
Not recorded	315 (19.0)	73 (21.7)	118 (16.9)	124 (20.0)	969 (21.3)	
BMI	31.42 ± 7.69	31.58 ± 7.71	31.67 ± 7.95	30.92 ± 7.36	28.65 ± 6.80	< 0.01
Smoking status						
Every day	266 (16.1)	65 (19.3)	100 (14.3)	101 (16.3)	657 (14.5)	< 0.01
Some day	51 (3.1)	15 (4.5)	16 (2.3)	20 (3.2)	207 (4.6)	
Not at all	540 (32.6)	97 (28.8)	237 (33.9)	206 (33.3)	869 (19.1)	
Not recorded	798 (42.8)	160 (47.5)	346 (49.5)	292 (47.2)	2806 (61.8)	
Direct HDL-Cholesterol (mg/dL)	53.65 ± 16.53	53.54 ± 15.94	54.83 ± 16.86	52.37 ± 16.41	52.80 ± 15.84	0.07
Overweight	852 (51.5)	165 (49.0)	379 (54.2)	308 (49.8)	1375 (30.3)	< 0.01
Total Cholesterol (mg/dL)	191.70 ± 43.01	190.77 ± 40.61	192.31 ± 44.33	191.53 ± 42.82	189.54 ± 40.19	0.08
Vitamin D (nmol/L)	72.11 ± 30.16	64.46 ± 28.91	76.77 ± 31.22	71.02 ± 28.66	62.96 ± 27.62	< 0.01
With FHD	859 (51.9)	180 (53.4)	340 (48.6)	339 (54.8)	1839 (40.5)	< 0.01
Diabetes	890 (25.1)	109 (32.3)	193 (27.6)	164 (26.5)	1839 (9.9)	< 0.01
Hypertension	999 (60.4)	208 (61.7)	435 (62.2)	356 (57.5)	1271 (28.0)	< 0.01

^a^ For continuous variables, numbers represent the mean ± standard deviation, and for categorical variables, numbers represent count (percentage)

Abbreviations: Direct HDL-Cholesterol, direct high-density lipoprotein-cholesterol; BMI, body mass index; RA, Rheumatoid arthritis; OA, Osteoarthritis; FHD, family history of diabetes

The serum concentrations of each of the three PFAAs and levels of two urine metabolites are shown in Table 3. The 6-year average PFNA, PFDA, and PFHxS concentrations were 0.90 ± 1.31 ng/mL, 0.31 ± 0.80 ng/mL, and 1.83 ± 2.29 ng/mL, respectively. Table 4 presents the associations between the serum concentrations of three types of PFAAs and arthritis subgroups. Individual serum levels of PFDA and PFHxS were strongly associated with RA. Additionally, individual PFDA serum levels were significantly correlated with other types of arthritis.

**Table 3:** Descriptive statistics 8-years average levels of perfluoroalkyl acids, urine albumin and urine creatinine

	Mean	SD	P25	P50	P75	IQR
**Perfluoroalkyl acids**						
PFNA (ng/ml)	0.90	1.31	0.40	0.70	1.10	0.70
PFDA (ng/ml)	0.31	0.80	0.10	0.20	0.30	0.20
PFHxS (ng/ml)	1.83	2.29	0.70	1.30	2.20	1.50
**Urine albumin (mg/L)**	48.96	314.38	4.30	8.50	17.80	13.50
**Urine creatinine (mg/dL)**	125.52	83.11	63.00	110.00	169.00	106.00

Abbreviations: P25, P50, P75, Lower, median and upper quartiles of variables; IQR, inter-quartile range

**Table 4:** Subgroup analysis of the association of arthritis subtype (RA, OA, and Others) and PFAAs

Subgroup^a^	With Disease	Without Disease	*P*-value
**RA**			
PFNA (ng/ml)	0.94 ± 0.83	0.89 ± 1.35	0.214
PFDA (ng/ml)	0.32 ± 0.84	0.28 ± 0.37	< 0.05
PFHxS (ng/ml)	2.04 ± 2.52	1.81 ± 2.26	< 0.05
**OA**			
PFNA (ng/ml)	0.90 ± 1.33	0.87 ± 0.73	0.513
PFDA (ng/ml)	0.31 ± 0.81	0.30 ± 0.35	0.708
PFHxS (ng/ml)	1.84 ± 2.30	1.77 ± 2.08	0.565
**Others**			
PFNA (ng/ml)	0.92 ± 0.99	0.89 ± 1.34	0.548
PFDA (ng/ml)	0.31 ± 0.83	0.28 ± 0.30	< 0.05
PFHxS (ng/ml)	1.96 ± 2.28	1.82 ± 2.29	0.130

^a^ For continuous variables, numbers represent the mean ± standard deviation

Abbreviations: RA, Rheumatoid arthritis; OA, Osteoarthritis; PFNA, perfluorononanoic acid; PFDA, perfluorodecanoic acid; PFHxS, perfluorohexane sulfonic acid; PFAAs, perfluoroalkyl acids

### Association between FHD and arthritis prevalence

**Fig. 2:**
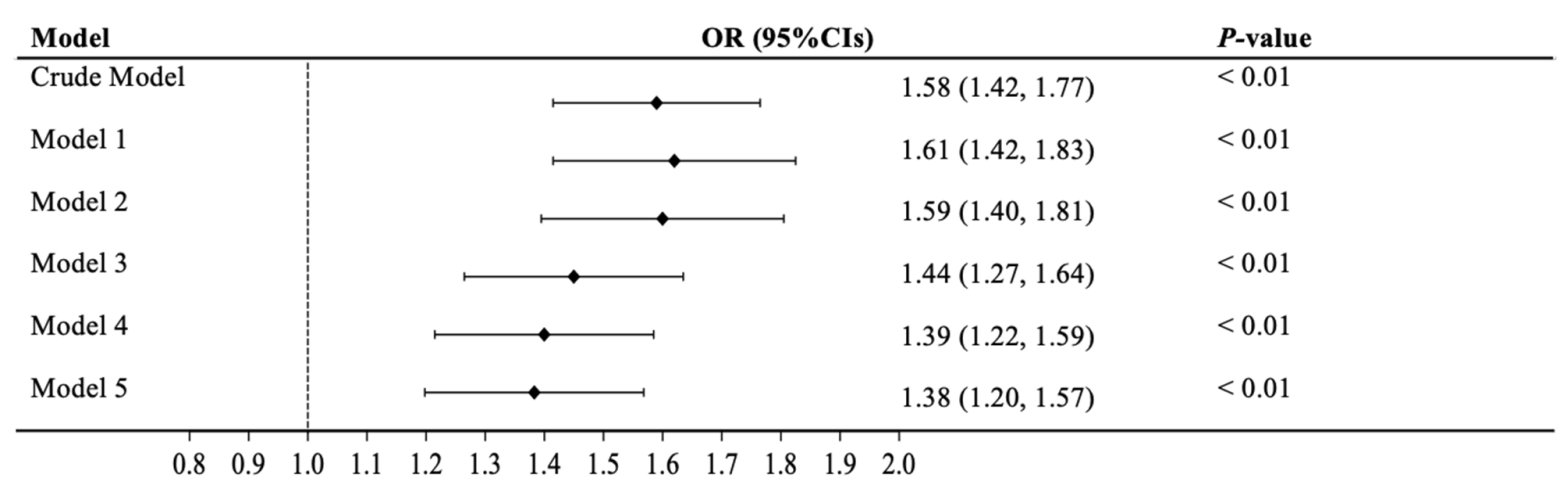
Association between the FHD and arthritis. Notes: Model 1, Adjusted for age, race, gender, and education; Model 2, Further adjusted for smoking and alcohol; Model 3, Further adjusted for BMI; Model 4, Further adjusted for diabetes; Model 5, Further adjusted for hypertension. FHD, family history of diabetes

**Fig. 3:**
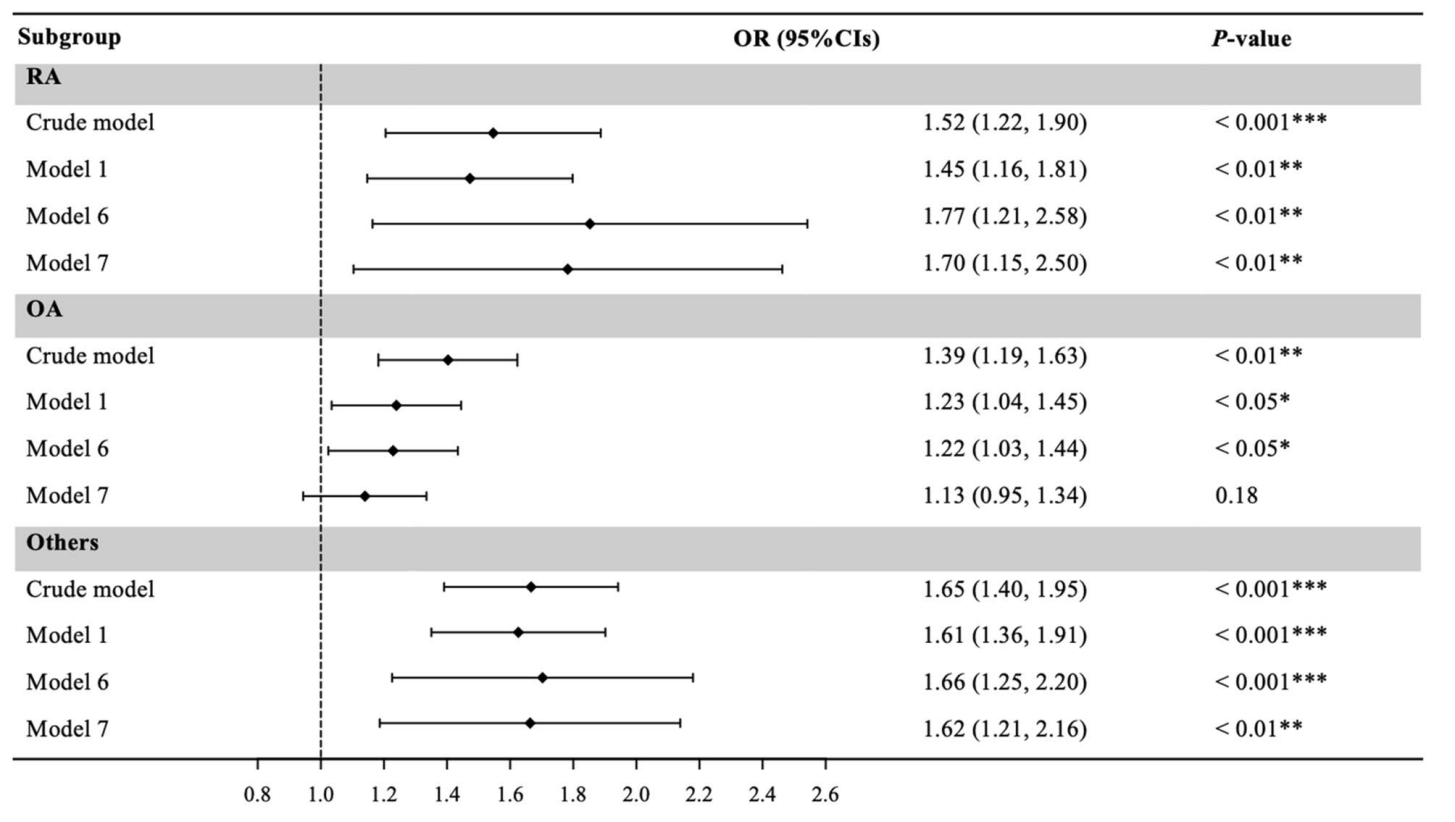
Subgroup analysis of the association of arthritis subtype (RA, OA, and Others) and FHD. Notes: Crude Model, without adjustment; Model 1, Adjusted for gender, age, race, and education; Model 6, Further adjusted for smoking, alcohol, and BMI; Model 7, Further adjusted for diabetes and hypertension. RA: rheumatoid arthritis; OA: osteoarthritis; FHD: family history of diabetes; *P < 0.05; **P < 0.01; ***P < 0.001

Figures 2 and 3 present associations between FHD and the prevalence of arthritis (Fig. 2) and the subgroups of arthritis (Fig. 3). FHD was associated with an increased incidence of arthritis in the crude model and the five adjusted models (Models 1, 2, 3, 4, and 5). FHD was also significantly correlated with RA and other types of arthritis in either the crude Model l or adjusted Models 1, 6, and 7.

After adjusting for age, race, sex, education, smoking status, alcohol status, BMI, diabetes, and hypertension, FHD was still strongly associated with both RA [OR = 1,70 (95% CI: 1.15–2.50)] and other types of arthritis [OR = 1.62 (95% CI: 1.21–2.16)]. In addition, the *P* value for the association between OA and FHD was 0.18, which was greater than the threshold value of *P* < 0.05, indicating a lack of a strong correlation after adjustment. However, there were significant associations between arthritis, RA, other types and FHD before and after adjustment.

### Association between PFAAs and arthritis and the effects of metabolism on the correlation of FHD with arthritis

As shown in Table 5, we first included PFAAs in the GLM models, revealing significant associations between PFNA and the risk of arthritis (Model 8). When FHD and one type of PFAAs were included in the same model, strong correlation was found between PFNA/PFDA and FHD on arthritis (Model 9). In Model 10, we involved an interaction term for FHD and each metabolic pathway. Significant associations were identified between PFDA and FHD and arthritis. The correlation between FHD and arthritis was strengthened with increased PFDA levels. However, statistically significant correlations were not detected between PFNA and FHD and arthritis (*P* = 0.203), demonstrating that PFNA does not strengthen the association between FHD and arthritis.

**Table 5:** Association between perfluoroalkyl acids and arthritis, and the modification effects of perfluoroalkyl acids in the association of FHD with arthritis

Model	X-arthritis		FHD-arthritis		*P*- interaction
	OR and 95%CI	*P*	OR and 95%CI	*P*	
**Model 8**					
PFNA (0.70 ng/ml)	1.36 (1.10, 1.69)	< 0.05*	-	-	-
PFDA (0.20 ng/ml)	1.14 (0.90, 1.38)	0.31	-	-	-
PFHxS (1.50 ng/ml)	1.22 (0.98, 1.52)	0.08	-	-	-
**Model 9**					
PFNA (0.70 ng/ml) + FHD	-	-	1.36 (1.10, 1.69)	< 0.05*	-
PFDA (0.20 ng/ml) + FHD	-	-	1.40 (1.13, 1.72)	< 0.05*	-
PFHxS (1.50 ng/ml) + FHD	-	-	1.20 (0.96, 1.50)	0.10	-
**Model 10**					
PFNA (0.70 ng/ml) + FHD + PFNA (0.70 ng/ml) × FHD	-	-	1.21 (0.96, 1.53)	0.10	0.203
PFDA (0.20 ng/ml) + FHD + PFDA (0.20 ng/ml) × FHD	-	-	1.33 (1.05, 1.68)	< 0.05*	0.023^**##**^
PFHxS (1.50 ng/ml) + FHD + PFHxS (1.50 ng/ml) × FHD	-	-	1.02(0.79, 1.30)	0.91	0.480

Notes: Model 8: univariate model, only perfluoroalkyl acids were applied in the model; Model 9: additive models, perfluoroalkyl acids, and an FHD were applied in the model, with perfluoroalkyl acids plus FHD; Model 10: interaction model, perfluoroalkyl acids and FHD were applied in the model, with perfluoroalkyl acids + FHD + perfluoroalkyl acids × FHD. *P < 0.05 for the association of FHD/ perfluoroalkyl acids and arthritis; ^**#**^*P* < 0.10; ^**##**^*P* < 0.05 for interactions of perfluoroalkyl acids and FHD.

Abbreviations: FHD, family history of diabetes

## Discussion

In this cross-national study of the 2011–2018 NHANES, we discovered that FHD is significantly correlated with an increased incidence of arthritis. Additionally, among all arthritis types, FHD mostly accounted for the increased prevalence of RA, followed by other types of arthritis (i.e., not RA or OA). We did not detect a significant correlation between FHD and OA after adjustment. We also observed that PFDA may promote the adverse impact of FHD on arthritis. To our knowledge, this is the first study to investigate the interactive effects of FHD and PFAAs on the risk of arthritis.

Our study revealed that PFDA levels could exacerbate the prevalence of arthritis in people with FHD. To our knowledge, no previous studies have tested the interactive effects of PFAAs on the correlation between FHD and arthritis. Nevertheless, several studies support our conclusions. First, some studies have revealed that people with FHD are more susceptible to diabetes, which is associated with PFAAs [20, 27, 28]. For example, a prospective case-control study revealed that after adjustment, a 60% higher risk prevalence of T2D was associated with plasma concentrations of PFOA and PFOS [28]. Second, the association between PFAAs and RA has been analyzed [29, 30]. For instance, a case-control study demonstrated that each IQR increase in PFOA exposure was associated with a 69% increase in the risk of RA [30]. Moreover, individuals with arthritis are more susceptible to diabetes and other metabolic diseases [11, 31, 32].

However, the mechanisms by which FHD affects arthritis remain poorly understood. Scientists have proposed several possible biological pathways for the relationship between diabetes and inflammatory diseases, including arthritis, cancer, depression, and systemic infections [33]. Several common mechanisms underlie diabetes and arthritis [31]. As a prevalent physiological mechanism in arthritis, chronic inflammation can lead to insulin resistance. Beta cells may be destroyed by higher serum pro-inflammatory cytokine levels [34–36]. FHD is a risk factor for diabetes development. Second, high blood sugar promotes the production of reactive oxygen species (ROS), molecules that promote the production of pro-inflammatory cytokines (secreted by certain immune cells). It also triggers the production of compounds called advanced glycation end products (AGEs), which accumulate in joints and cause damage, eventually resulting in arthritis [37]. Another important mechanism involves drugs used to treat arthritis. Prednisone and other steroids increase blood sugar levels by stimulating the liver to release more glucose and slow its movement to muscle and adipose tissue, which may lead to diabetes [38]. These mechanisms are consistent with our findings; we observed significant correlations between FHD and PFAAs and a joint effect of PFAAs on the relationship between FHD and arthritis.

Our study had several strengths. First, this is the first study to examine the association between FHD and arthritis, thus contributing epidemiological evidence to the field of arthritis research. In addition, we found that PFAA exposure increases the risk of developing arthritis. The main pathway by which the human body absorbs PFAAs is the intake of contaminated water or food [39]. Therefore, it is crucial to detect PFAAs in food and to perform rigorous toxicological assessments of chemicals discharged into water to reduce exposure, which requires the joint efforts of the World Health Organization and every nation. Additionally, we included a model with interaction effects in this study. Different from common studies on the correlation between the two diseases, our use of an interaction model provides additional insight into the impact of PFAAs, a common contaminant, on the relationship between diabetes and FHD. It is important to raise awareness about the adverse effects of PFAAs and to reduce the use of these chemicals. Finally, precise calculations and robust support from the literature suggest that our results are reliable.

Our study also had several limitations. As a cross-sectional survey, the NHANES cannot provide longitudinal follow-up data. Further research is required to establish a causal relationship between FHD and arthritis. In addition, some characteristics were not included in the NHANES, such as CRP, salt intake, and low-density lipoprotein (LDL) for 2015–2018. As an inflammatory marker substance, CRP is elevated in some patients with arthritis. However, recent research does not support a significant relationship between CRP and arthritis [40, 41]. A previous study has also found that excessive salt intake is correlated with the development of RA [42]. However, the study population included individuals with various salt intake patterns. LDL levels are also elevated in patients with arthritis [43]. If these factors are added to the analysis, we expect the OR value to decrease slightly, without influencing the final results. In the future, it may be useful to select years in which data for these two parameters are available for a shorter-term study. Moreover, diseases in the NHANES are diagnosed through self-reported questionnaires, resulting in unavoidable bias; therefore, the conclusions need further verification. Furthermore, the concentrations of PFAAs changed over time; however, participants were only evaluated at a single time point and, therefore, the results do not fully reflect exposure to PFAAs. Finally, owing to insufficient data, PFOA and PFAS were not included in the analyses.

## Conclusion

Our study showed that FHD is associated with a higher prevalence of arthritis. The adverse effects of FHD on arthritis are exacerbated by increased PFDA levels in American adults. Thus, immediate preventive measures should be undertaken to reduce PFAAs exposure and prevent arthritis in people with FHD.

## Data Availability

The datasets generated and analysed during the current study are available in the National Health and Nutrition Examination Survey repository, https://wwwn.cdc.gov/nchs/nhanes/Default.aspx.
